# When pain becomes self: limbic-default mode network hyperconnectivity predicts microvascular decompression failure in trigeminal neuralgia

**DOI:** 10.1093/braincomms/fcag220

**Published:** 2026-06-11

**Authors:** Ying Wang, Chenglong Cao, Hao Chen, Min Wu, Xiaosong He, Xiaofeng Jiang

**Affiliations:** Department of Neurosurgery, The First Affiliated Hospital of USTC, Division of Life Sciences and Medicine, University of Science and Technology of China, Hefei, Anhui Province 230001, P. R. China; Anhui Provincial Stereotactic Neurosurgical Institute, Hefei, Anhui Province 230001, P. R. China; Anhui Province Key Laboratory of Brain Function and Brain Disease, Hefei, Anhui Province 230001, P. R. China; Department of Neurosurgery, The First Affiliated Hospital of USTC, Division of Life Sciences and Medicine, University of Science and Technology of China, Hefei, Anhui Province 230001, P. R. China; MRC Brain Networks Dynamics Unit, University of Oxford, Oxford, UK; Department of Radiology, The First Affiliated Hospital of USTC, Division of Life Sciences and Medicine, University of Science and Technology of China, Hefei, Anhui 230001, China; Department of Neurosurgery, The First Affiliated Hospital of USTC, Division of Life Sciences and Medicine, University of Science and Technology of China, Hefei, Anhui Province 230001, P. R. China; Department of Psychology, University of Science and Technology of China, Hefei, Anhui Province 230026, China; Department of Neurosurgery, The First Affiliated Hospital of USTC, Division of Life Sciences and Medicine, University of Science and Technology of China, Hefei, Anhui Province 230001, P. R. China

**Keywords:** trigeminal neuralgia, microvascular decompression, pain embedding, default mode network, functional connectivity

## Abstract

Microvascular decompression is the standard surgical intervention for classical trigeminal neuralgia attributed to NVC. However, up to 30% of patients experience poor outcomes despite technically successful surgery, suggesting non-peripheral mechanisms. This study investigates whether central neural reorganization—manifested as altered cortico-subcortical connectivity and white matter microstructure—underlies poor surgical outcomes in patients with ‘incidental’ low-pressure vascular contact. In this prospective nested case-control study, we enrolled 60 patients with medication-refractory classical trigeminal neuralgia who underwent microvascular decompression and 30 matched healthy controls. Based on the 3-month surgical outcome, patients were retrospectively stratified into effective and ineffective groups. All participants underwent preoperative resting-state functional MRI and diffusion tensor imaging. Whole-brain functional connectivity between 100 cortical (Schaefer atlas) and 16 subcortical (Tian atlas) regions was analysed. Connections were ranked by between-group effect size (Cohen’s *d*); the top 10 were used to train a SVM classifier. Correlational tractography assessed the relationship between fractional anisotropy and disease duration. Despite comparable preoperative pain, the ineffective group had significantly lower intraoperative vascular pressure (*P* = 0.02). Compared to the effective group, the ineffective group exhibited significantly enhanced functional connectivity between default mode, somatomotor and control networks and subcortical limbic structures (amygdala, nucleus accumbens, hippocampus) (*P* < 0.05, permutation test). A SVM classifier trained on these features achieved 86.0% accuracy (AUC = 0.931) in distinguishing outcome groups. Diffusion tensor imaging analysis revealed that longer disease duration was associated with decreased fractional anisotropy in sensorimotor white matter tracts, indicating progressive microstructural decline. Poor surgical outcome is not merely ineffective decompression, but a signature of maladaptive central reorganization—specifically, heightened default mode network–limbic coupling. This hyperconnectivity indicates that pain has become cognitively and affectively ‘self-embedded’, transitioning from a sensory event to a persistent self-referential narrative that sustains pain even after peripheral trigger removal. These results reframe a subset of trigeminal neuralgia as a central network disorder of pain self-representation and identify default mode network–amygdala hyperconnectivity as a candidate target for circuit-based neuromodulation—such as repetitive transcranial magnetic stimulation over medial prefrontal hubs—to ‘unlearn’ the embedded pain trace, either adjunctively or as an alternative to microvascular decompression.

## Teaser

Wang *et al*. report that microvascular decompression failure in trigeminal neuralgia is associated with heightened default mode-limbic connectivity. This ‘pain embedding’ signature—where pain integrates into self-representational circuits—predicts surgical outcomes with high accuracy and suggests that some patients may benefit from neuromodulation rather than peripheral decompression.

## Graphical Abstract

**Graphical Abstract:**
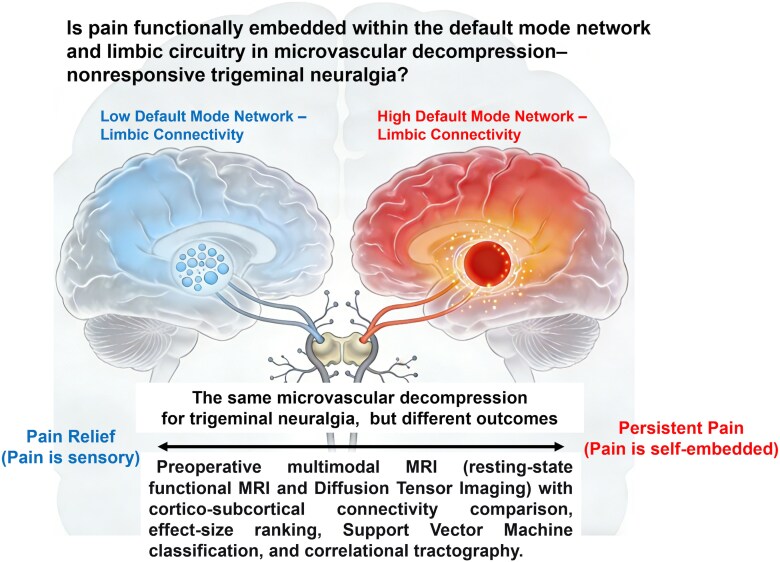

## Introduction

Trigeminal neuralgia (TN) is classically attributed to neurovascular compression (NVC) at the trigeminal root entry zone. For medically refractory cases, microvascular decompression (MVD) is the most effective and preferred surgical intervention, offering superior long-term pain relief with minimal sensory morbidity compared to neurodestructive procedures.^1,2^

However, a persistent clinical paradox exists: a substantial proportion of patients—including those with clear radiographic evidence of vascular contact—experience suboptimal or poor outcomes following MVD.^3,4^ This discrepancy cannot be fully explained by the anatomical severity of compression alone,^5^ strongly suggesting that other, potentially central mechanisms underlie treatment resistance.

Our recent haemodynamic investigations have provided a crucial clue to this paradox. We identified a distinct subgroup of TN patients in whom the offending vessel exhibited low pulsatile pressure despite apparent imaging contact.^6^ This finding challenges the long-held assumption that all radiographically visible neurovascular conflicts are functionally pathogenic. Instead, it introduces the concept of ‘incidental’ or non-pathogenic vascular contact—a ‘vascular illusion’ that may coexist with, but not directly drive, the painful syndrome.^5^ This subgroup likely accounts for a significant portion of MVD non-responders.

This observation raises a pivotal, unanswered question: if not persistent peripheral compression, what sustains TN in these patients? We hypothesize that maladaptive central sensitization—manifesting not only in the classic thalamo-somatosensory pathway but more dominantly within higher-order circuits governing the affective and cognitive dimensions of pain—is the key sustaining mechanism. In classical pain processing, trigeminal nociceptive signals are relayed via the thalamus to the primary somatosensory cortex (S1), which encodes the sensory-discriminative aspects of pain.^7,8^ Prolonged nociceptive input can induce neuroplastic changes in this pathway, leading to heightened thalamocortical connectivity—a hallmark of central sensitization.^9,10^ Critically, the limbic system (e.g. amygdala, hippocampus) and the default mode network (DMN) encode the emotional salience and self-referential evaluation of pain, respectively. Hyperconnectivity between these circuits may represent a self-sustaining central state in which pain is no longer a direct sensory readout but has become embedded in emotional and cognitive constructs.^11^ Such central reorganization can persist independently of peripheral triggers, offering a compelling explanation for why MVD fails when the true pathology is central.

Prior neuroimaging studies provide indirect support for central involvement, showing that poor MVD outcomes correlate with volumetric alterations in subcortical pain-related structures.^12^ Furthermore, established central sensitization may endure even after successful surgical decompression.^11^ However, a critical gap remains: no study has directly examined whether preoperative dysfunction of the cortico-limbic circuits—particularly in patients with incidental vascular contact—predicts surgical resistance. Identifying such a preoperative biomarker could transform patient selection and personalize treatment strategies.

To address this gap, we conducted a multimodal neuroimaging study integrating resting-state functional MRI (rs-fMRI) and diffusion tensor imaging (DTI). We recruited three well-matched cohorts: TN patients with effective MVD outcomes, those with ineffective outcomes and healthy controls (HCs). Our aims were 3-fold:

1. To test the hypothesis that patients with poor MVD outcomes exhibit enhanced functional connectivity, particularly between higher-order cortical networks (e.g. DMN) and limbic subcortical structures, indicative of maladaptive central sensitization
2. To determine the efficacy of these connectivity patterns in discriminating between patients with effective versus ineffective outcomes, using a machine-learning [support vector machine (SVM)] framework for objective classification
3. To examine white matter microstructural integrity [via fractional anisotropy (FA)] in sensorimotor pathways, exploring whether functional alterations coincide with structural decline—a signature of maladaptive neuroplasticity

Collectively, these aims serve a broader conceptual objective: to test whether a subset of TN represents not only a peripheral compressive syndrome but also a central network disorder in which pain has become functionally embedded within the neural circuitry of self-representation. If confirmed, this framework would redefine poor surgical outcome not as surgical failure, but as disease misclassification—and would support the use of preoperative fMRI as a phenotyping tool to guide mechanism-based treatment selection.

## Materials and methods

### Participants

We prospectively recruited patients with unilateral classical TN undergoing MVD at our institute between May 2022 and December 2023. From the enrolled cohort, we adopted a nested case-control design: based on 3-month postoperative outcomes, we identified 30 patients with ineffective MVD outcomes (cases) and selected 30 age- and sex-matched patients with effective outcomes (controls) for neuroimaging analysis. Thirty age- and sex-matched HCs were also enrolled. Demographic characteristics are summarized in Table 1.

**Table 1:** Demographic data of the participants

	E	NE	HC	*P*-value
*N*	30	30	30	
Age	58.16 ± 8.17	58.22 ± 6.61	60.13 ± 6.38	0.18
Gender	18 females	18 females		1.00
Duration	5.03 ± 3.47	5.81 ± 4.42		0.44
painful side	3 left	5 left		0.45
HAMA score	12.07 ± 3.41	12.10 ± 1.96		0.91
HAMD score	13.37 ± 4.81	13.17 ± 1.96		0.53
VAS score before MVD surgery	9.9 ± 0.40	9.81 ± 0.53		0.47
Pressure (Pa)	37.29 ± 21.88	23.63 ± 15.92		0.02
Postoperative VAS scores	0 ± 0	5.72 ± 1.40		<0.001

HAMA, Hamilton Anxiety; HAMD, Hamilton Depression; VAS, visual analogue scale; MVD, microvascular decompression.

Participants comprised consecutive patients with medication-refractory classical TN confirmed through preoperative neurological evaluation and MRI/MRA by senior neurosurgeons. Diagnosis was strictly based on the International Headache Society (IHS) criteria, which mandate documented paroxysmal electric shock-like facial pain within the trigeminal dermatomes, objective neurovascular conflict on imaging and responsiveness to anticonvulsants prior to treatment failure. All enrolled patients were undergoing MVD as their first surgical or interventional procedure for TN; those with a prior history of any TN-related surgery (e.g. radiofrequency thermocoagulation, glycerol rhizotomy, balloon compression or stereotactic radiosurgery) or peripheral nerve block/ablation were excluded. Surgical candidates exhibited an ASA I-II physical status, with radiologically verified vascular compression and absent confounding pathologies. Then, they underwent MVD, with the procedure strictly adhering to the Chinese Expert Consensus on the Diagnosis and Treatment of Trigeminal Neuralgia, issued by the Neurosurgery Branch of the Chinese Medical Association, and referencing internationally recognized technical standards. Exclusion criteria systematically eliminated cases with persistent interparoxysmal pain, atypical symptomatology, adequate medication response, coagulopathy, insufficient imaging evidence or comorbidities that contraindicated craniotomy, ensuring cohort homogeneity for central mechanism analyses.

All MVD procedures were performed via a standard retrosigmoid approach. The offending vessel was carefully dissected from the trigeminal nerve and transposed, with a small Teflon pledget placed between the vessel and the nerve root to maintain decompression. In no case were additional destructive procedures—such as internal neurolysis, partial sensory rhizotomy or nerve section—performed. This consistent surgical approach ensures that outcome differences reflect biological response to decompression rather than procedural variation.

All participants were right-handed with no significant medical illnesses, major psychiatric disorders, or neurological illnesses, or displayed gross structural abnormalities based on their T_1_-weighted images. No participants had a history of dependence (current or past) on any drug. Informed consent was obtained from all participants, and the study was approved by the local institutional review board, conforming to the tenets of the Declaration of Helsinki.

### Clinical evaluation and grouping

All patients were assessed using a Visual Analogue Scale (VAS), the Hamilton Depression Scale and the Hamilton Anxiety Scale prior to surgery, as well as one day and three months postoperatively. CTN patients whose pain disappeared immediately after surgery and had no recurrence within one year were included in the effective group (E group). CTN patients whose pain did not completely disappear after surgery and had pain within three months were included in the non-effective group (NE group). All patients used carbamazepine preoperatively at a dose of 0.2 g three times daily, with a maximum daily dose of 1.2 g.

### MRI data acquisition

Gradient-echo planar magnetic resonance imaging data were obtained 1 day before and after surgery on a 3.0 T GE MR750w (USA) at our institute. Before entering the MRI scanner, all participants were instructed to keep their heads still with their eyes closed during all scans. A circularly polarized head coil was used, with foam padding to restrict head motion. Resting-state MRI data with 242 frames were acquired with a T_2_*-weighted echo-planar imaging sequence (TE = 30 ms, TR = 2000 ms, FOV = 240 mm, matrix = 64 × 64, flip angle = 85°) with 33 axial slices (no gaps, voxel size: 3.6 × 3.6 × 3.6 mm^3^) covering the whole brain. Corresponding high-resolution T_1_-weighted three-dimensional gradient-echo (for stereotaxic transformation) images were also collected [TR = 1900 ms; TE = 2.26 ms; TI = 900 ms; 1 mm isotropic voxel; 250 mm field of view (FOV)].

### fMRI data analysis

All analyses of MRI data were conducted on a computing system running the Ubuntu 24.04 Linux operating system. Data from all three groups were converted to Brain Imaging Data Structure (BIDS) format following the established tutorial. This conversion process involved the installation of Miniconda as a prerequisite, followed by the installation and execution of the dcm2bids tool.

### Resting-state fMRI data analysis

**Figure 1:**
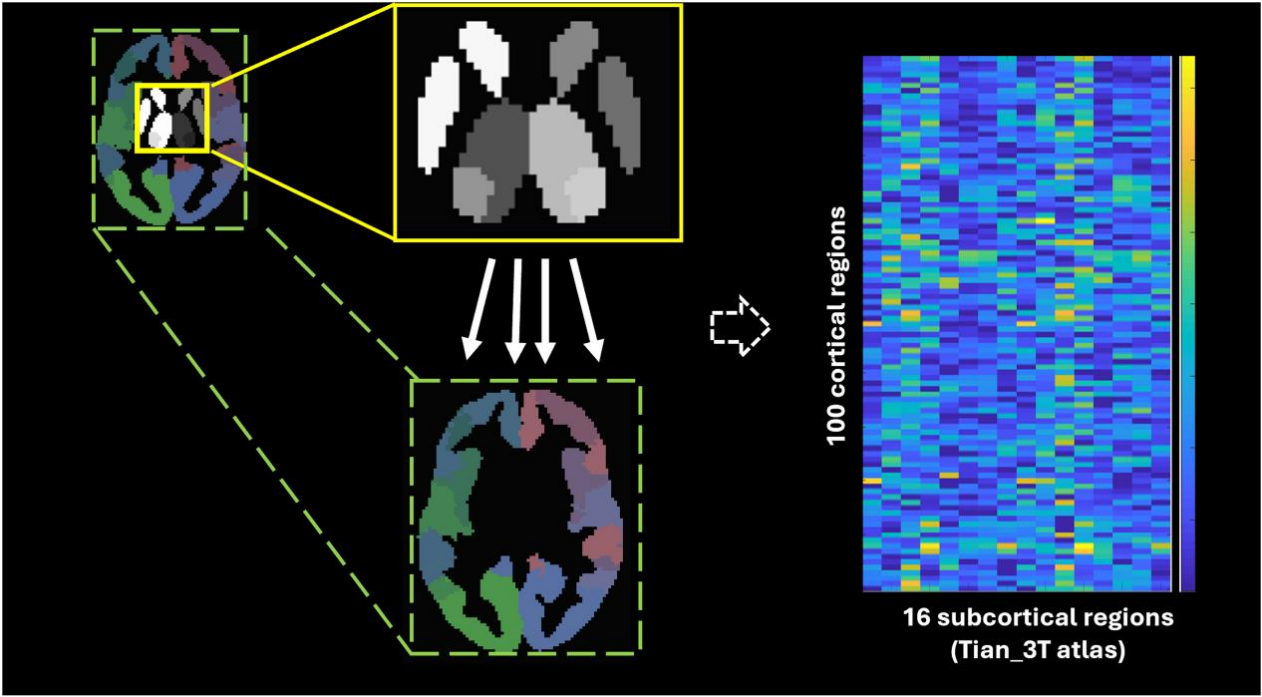
**Schematic of the resting-state fMRI data analysis pipeline.** The pipeline depicts key processing stages: preoperative fMRI data acquisition, preprocessing with fMRIPrep and xcp_d, time series extraction from 100 cortical (Schaefer atlas) and 16 subcortical (Tian atlas) regions, construction of functional connectivity matrices via Pearson correlation and retention of the top 10% strongest connections for subsequent group comparisons. fMRI, functional magnetic resonance imaging; MVD, microvascular decompression; E, effective outcome group; NE, ineffective outcome group; HC, healthy control.

Results included in this manuscript stem from preprocessing conducted with fMRIPrep 24.0.0,^13^ which operates on Nipype 1.8.6.^14^ Following preprocessing, we initially employed the preprocessed fMRI data to quantify head movements and other confounding variables, subsequently utilizing these estimates in Xcp-d to denoise the fMRI signal and assess functional connectivity. The Schaefer 100 parcels scheme constitutes a sophisticated framework for delineating cerebral cortex regions, categorizing them into 100 distinct areas grounded in extensive neuroimaging data and advanced analytical methodologies.^15^ Hence, we extracted the time series of resting-state fMRI data from 100 brain regions of interest (ROIs) in the Schaefer Atlas. Furthermore, using the Tian_3T atlas (comprising 16 subcortical regions)^16^ as a template, we extracted the mean time series from each subcortical region. Subsequently, we computed Pearson correlation coefficients between all pairs of these time series to construct functional connectivity matrices (the pipeline of the analysis is shown in Fig. 1).

### Haemodynamic parameter computation (computational fluid dynamics)

The methodology for this section references our previously published work.^6^ To objectively quantify the haemodynamic impact of the offending vessels in a subgroup of patients, computational fluid dynamics (CFD) simulations were performed based on preoperative Time-of-Flight Magnetic Resonance Angiography (TOF-MRA) images, following a recently established and validated pipeline.^6^ The offending vessel and NVC site were identified and reconstructed from MRA images using RadiAnt DICOM Viewer (Medixant) and Mimics Research (Materialise). The three-dimensional geometry was then smoothed in Geomagic Wrap (3D Systems) and meshed in ANSYS Fluent Meshing. After applying physiologically realistic boundary conditions (pulsatile velocity inlet, Windkessel-model pressure outlets), the governing Navier-Stokes equations were solved using ANSYS Fluent to simulate blood flow. Key haemodynamic parameters were extracted from the NVC zone, including peak systolic flow (PSF) and maximum wall shear stress (Max WSS), which have been identified as significant predictors of MVD outcome.^6^ Patients were categorized into subgroups with distinct haemodynamic profiles (e.g. high versus low vascular pressure/flow patterns) based on these CFD-derived metrics for subsequent correlation with central imaging findings and clinical outcomes.

### Statistical analysis

#### Demographical, behavioural and clinical comparisons

To avoid the confounding effects of factors such as age, sex, disease duration, painful side, anxiety and depression levels, we analysed and compared the differences in disease duration, age and sex between the E and NE groups with two-sample *t*-tests or *χ*² test (Table 1). Furthermore, two-sample *t*-tests were employed to compare preoperative VAS scores, the haemodynamic parameter (pressure, Pa) and postoperative VAS scores between the E and NE groups.

#### Permutation test for connectivity strength

This study focused on interregional connectivity between the subcortical and cortical regions. This study employed permutation testing to statistically compare brain functional connectivity matrices between two groups. The experimental group (E-group) and control group (NE-group) each consisted of 30 participants, with data represented as connection strength matrices between 100 cortical and 16 subcortical regions (100 × 16 × 30 per group). The analytical procedure comprised the following key steps:

First, each subject's connectivity matrix was flattened into a 1600-dimensional vector, and group-level data matrices were constructed. Two-sample *t*-tests were performed for each connection to compute the true between-group differences, yielding t-statistics, *P*-values and Cohen's d effect sizes.

Subsequently, permutation testing was conducted with 5000 permutations. In each permutation, group labels were randomly shuffled, and t-statistics were recalculated for all connections to build a permutation distribution. Based on this distribution, two-tailed permutation *P*-values were computed for each connection. Family-wise error (FWE) correction was applied using the maximum statistic method to control for multiple comparisons.

Furthermore, false discovery rate (FDR) correction was applied to the permutation *P*-values, with a threshold set at *q* < 0.05. Connections surviving this threshold were deemed statistically significant.

Finally, results for significant connections—including t-values, corrected *P*-values and effect sizes—were output. Visualization of results was performed using heatmaps, permutation distribution histograms and other graphical representations. All analyses were implemented in the MATLAB environment. Given the conservative nature of FWE/FDR correction in high-dimensional connectivity data, we additionally performed an effect-size-based ranking analysis to identify candidate features for hypothesis generation and machine learning.

### Effect size ranking analysis

To quantify the magnitude of differences in cortico-subcortical functional connectivity between the experimental (E) and control (NE) groups, Cohen's *d* effect size was employed as the primary metric. The calculation procedure was conducted as follows: for each connection between cortical region i (i = 1–100) and subcortical region j (j = 1–16), Cohen’s d was calculated as


$$d_{ij}=\frac{{\bar{X}}_{E,ij}-{\bar{X}}_{NE,ij}}{s_{pooled,ij}}$$

where

-
${\bar{X}}_{E,ij}$ and ${\bar{X}}_{NE,ij}$ represent mean connectivity strength for E and NE groups, respectively.
-
$s_{pooled,ij}$ denotes the pooled standard deviation:
  $$s_{pooled,ij}=\sqrt{\frac{(n_{E}-1)s_{E,ij}^{2}+(n_{NE}-1)s_{NE,ij}^{2}}{n_{E}+n_{NE}-2}}$$
-
$s_{E,ij}$ and $s_{NE,ij}$ are standard deviations for each group.
-
$n_{E}=n_{NE}=30$ indicates sample size per group.

**Figure 2:**
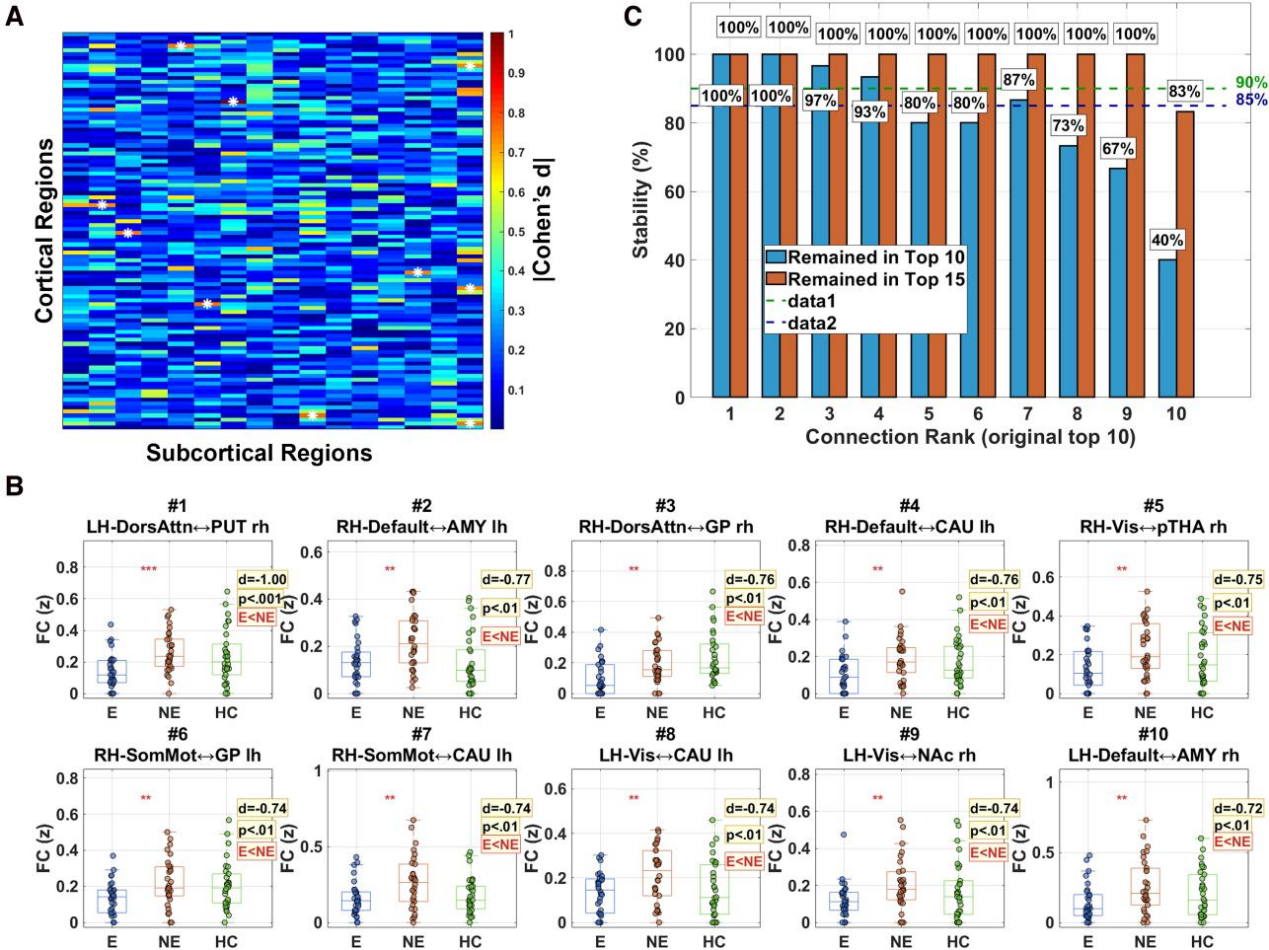
**Top 10 functional connections with largest effect sizes between groups.** (**A**) Heatmap showing Cohen’s *d* effect size for differences in functional connectivity between the ineffective (NE) and effective (E) MVD outcome groups (*N* = 30 per group). Positive values (blue) indicate stronger connectivity in the E group; negative values (orange) indicate stronger connectivity in the NE group. White asterisks mark the top 10 connections by absolute effect size. (**B**) Box plots (mean ± SEM) showing Fisher’s z-transformed functional connectivity strength for the 10 connections with the largest effect sizes. Each data point represents an individual subject (*N* = 30 per group for E, NE and HC). Statistical significance between E and NE groups was assessed using permutation testing with 5000 iterations (*P < 0.05, **P < 0.01, ***P < 0.001). All 10 connections demonstrated stronger connectivity in the NE group versus E group. Note the consistent positioning of HC intermediate between the E and NE groups, with NE exhibiting the highest connectivity strength. Subcortical region abbreviations: HIP, hippocampus; AMY, amygdala; pTHA, posterior thalamus; aTHA, anterior thalamus; NAc, nucleus accumbens; GP, globus pallidus; PUT, putamen; CAU, caudate; -rh, right hemisphere; -lh, left hemisphere. (**C**) Leave-one-out sensitivity analysis. Bar plot showing the percentage of iterations (*N* = 60 iterations, each excluding one subject) in which each of the original top 10 connections remained within the top 10 (blue) or top 15 (orange) after recalculating effect sizes. The four highest-ranked connections showed >93% stability in the top 10, and all connections remained in the top 15 in ≥83% of iterations (average: 98.3%). Dashed lines indicate 90% and 85% thresholds.

To identify the most clinically relevant connectivity differences, systematic ranking analyses were performed. All 1600 connections were ranked by absolute effect size in descending order, and the significant top 10 connections were then used as features in a SVM model. The goal of this machine learning step was to assess the reliability and stability of these differences in a predictive context for group classification. The 10 connections with the largest effect sizes were visualized in detail (Fig. 2). To contextualize the differences observed between the E and NE groups within a normative framework, we specifically examined the functional connectivity values of the HC group in the same connections identified as having the largest effect sizes (Cohen’s *d*) from the E versus NE comparison. For each of these top connections, we extracted the mean Fisher’s z-transformed correlation coefficient for the HC group. This allowed for a direct, quantitative comparison of the group means across all three groups (E, NE and HC) to determine their relative positions (e.g. E < HC < NE, HC < E < NE, etc.). This tri-group analysis aimed to clarify whether the NE group’s connectivity profile was closer to the HC baseline or represented an intermediate or divergent state relative to both the E and HC groups.

To verify that the top-ranked connections were not disproportionately influenced by individual outliers, we performed a leave-one-out sensitivity analysis. In each of 60 iterations, one subject was excluded, Cohen’s *d* was recalculated for all 1600 connections using the remaining 59 subjects, and connections were re-ranked by absolute effect size. We then recorded whether each of the originally identified top 10 connections remained within the top 10 or top 15 of each iteration’s new ranking.

### Network-level effect size analysis

**Figure 3:**
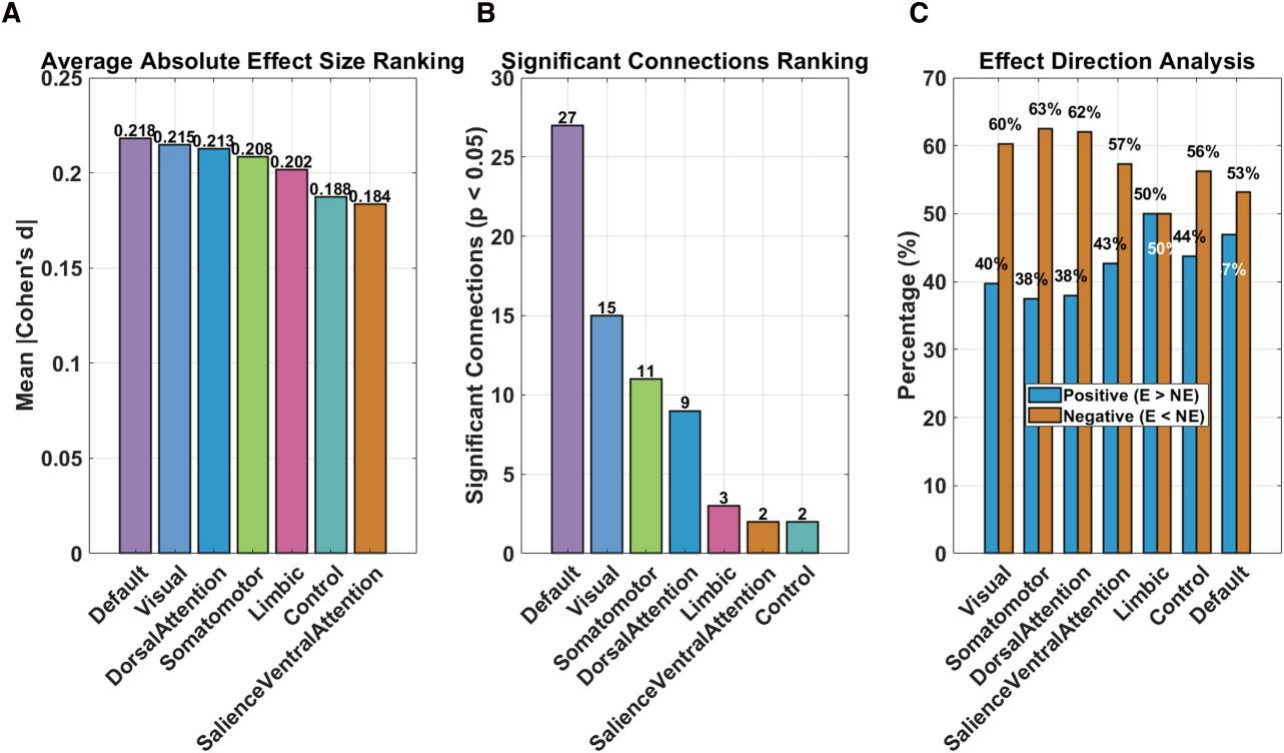
**Network-level effect size analysis of cortico-subcortical connectivity differences.** (**A**) Bar chart displaying mean absolute effect size (|Cohen’s *d*|) for seven large-scale functional networks, ranked in descending order. Numbers above bars indicate the number of significant connections (permutation test with 5000 iterations, *P* < 0.05) within each network. The Default network showed the highest mean |d| (0.228), followed by Somatomotor (0.219), Limbic (0.205), Visual (0.202), Salience/Ventral Attention (0.195), Control (0.193) and Dorsal Attention (0.185) networks. Error bars represent standard error of the mean (SEM). (**B**) Bar chart showing the number of significant connections (permutation test *P* < 0.05) for each functional network. The Default network exhibited the highest count (27 significant connections), followed by Somatomotor (21), Visual (19), Dorsal Attention (18), Limbic (13), Control (4) and Salience/Ventral Attention (4) networks. (**C**) Stacked bar chart showing the proportion of connections within each functional network where connectivity strength was higher in the effective group (E > NE, blue) versus higher in the ineffective group (E < NE, orange). Across all networks, the majority of connections showed E < NE pattern, indicating stronger connectivity in the NE group, consistent with widespread hyperconnectivity in the poor-outcome group.

Based on standardized anatomical labels derived from the Schaefer 2018 brain atlas, each of the 100 cortical regions was systematically assigned to one of seven large-scale functional networks: the Visual network, Somatomotor network, Dorsal Attention network, Salience/Ventral Attention network, Limbic network, Control network or Default Mode network. For each functional network, several network-level effect size metrics were computed—including mean absolute effect size (average of |Cohen's d| across connections), mean directional effect size (arithmetic mean of Cohen's d indicating overall direction of group differences), number of significant connections (count of connections with permutation test *P* < 0.05) and maximum effect size (largest |Cohen's d| within the network)—and networks were then ranked by mean absolute effect size in descending order to identify those most prominently involved in between-group differences (Fig. 3).

### Model construction

**Figure 4:**
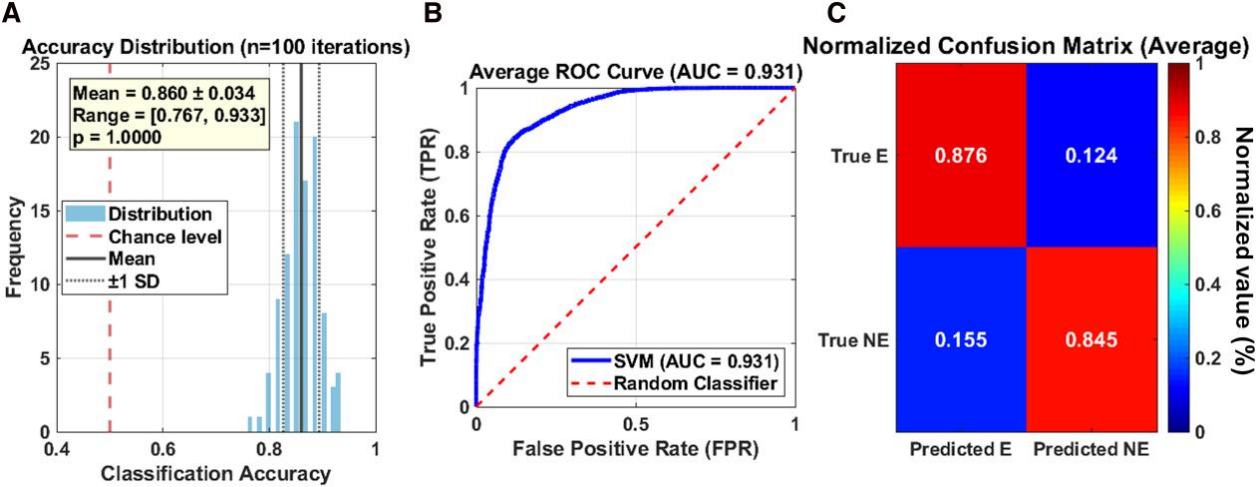
**Performance of SVM classifier in predicting MVD outcome.** (**A**) Summary of classification performance metrics across 100 random train-test splits (mean ± SD). The classifier was trained using the top 10 functional connections as features with a RBF kernel. *N* = 60 total subjects (30 E, 30 NE), with 80% used for training (*N* = 48) and 20% for testing (*N* = 12) in each iteration. The model achieved a mean accuracy of 86.0% ± 3.4% and a mean AUC of 0.931 ± 0.017, with balanced precision, recall and specificity. (**B**) Mean ROC curve across all cross-validation iterations. Shaded area represents ±1 standard deviation. The AUC is 0.931 ± 0.017 (chance level: 0.5, dashed diagonal), indicating excellent discriminative ability. *x*-axis: false positive rate (1—specificity); *y*-axis: true positive rate (sensitivity). (**C**) Average normalized confusion matrix aggregated over 1200 predictions (12 test subjects × 100 iterations). Values represent mean percentages with standard deviations in parentheses. The classifier correctly identified 86.9% of effective-outcome patients and 85.1% of ineffective-outcome patients.

A random subset comprising 80% of the patients (48 out of 60) was assigned to the training set, with the remaining 20% constituting an independent test set. A SVM model with a Gaussian radial basis function (RBF) kernel was constructed using the top 10 significant functional connections. To ensure robust performance evaluation, we conducted 100 random training-test splits and model trainings, resulting in a total of 1200 data points (12 subjects × 100 tests). For each iteration, the model’s performance was evaluated on the corresponding test set, using assessment metrics that included classification accuracy, true positive/negative rates, false positive/negative rates, precision, recall, specificity and area under the curve (AUC). The average performance across all 100 iterations was reported to provide a stable estimate of model generalizability. The classification performance was further visualized using average confusion matrices and receiver operating characteristic (ROC) curves. All analyses were performed using MATLAB 2018b (Fig. 4).

### DTI analysis

DTI analysis was conducted with DSI Studio (2024.05.22 ‘Chen’ Release). A total of 60 diffusion MRI scans were included in the connectometry database. A DTI diffusion scheme was used, and a total of 30 diffusion sampling directions were acquired. The b-value was 1000 s/mm^2^. The in-plane resolution was 0.875 mm. The slice thickness was 2.50 mm. The diffusion MRI data were rotated to align with the AC-PC line at an isotropic resolution of 2 (mm). The restricted diffusion was quantified using restricted diffusion imaging.^17^ The diffusion data were reconstructed using generalized q-sampling imaging with a diffusion sampling length ratio of 1.25. The tensor metrics were calculated using DWI with a b-value lower than 1750 s/mm^2^. The dti_fa values were used in the connectometry analysis.

**Figure 5:**
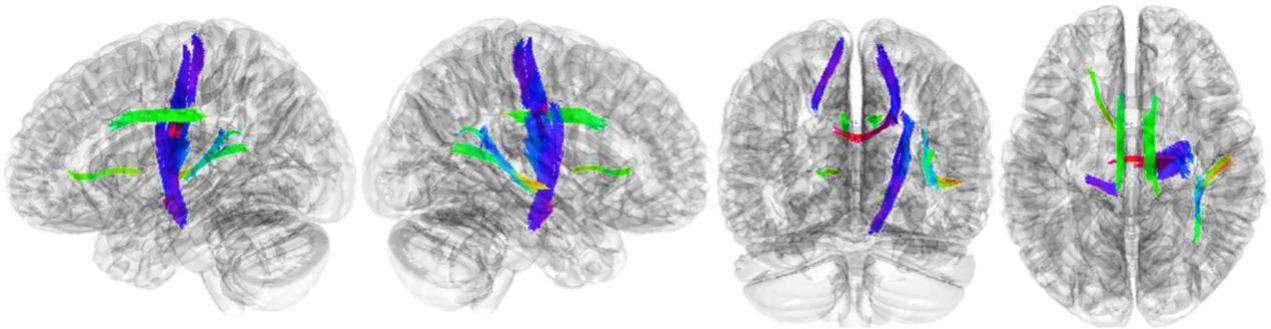
**Correlational tractography showing white matter tracts where FA correlates with disease duration.** Tracks demonstrate a negative correlation between FA and disease duration, indicating microstructural decline in pathways connecting subcortical to somatosensory motor regions with longer illness duration. Statistical significance was assessed using Spearman’s correlation with permutation testing (4000 randomizations, FDR < 0.05, T-score threshold = 2.5). *N* = 60 patients. Tracks are colour-coded by the strength of the negative correlation (Spearman’s ρ). Age and disease duration were not correlated in this sample (Pearson’s *r* = 0.032, *P* = 0.808), suggesting that observed microstructural alterations are unlikely to be driven by normal aging alone. Colour coding indicates the strength of the negative correlation (Spearman’s ρ) between FA and disease duration, ranging from weakest (cool colours, dark blue) to strongest (warm colours, red). Blue tracts represent weaker negative correlations, and red tracts represent stronger negative correlations. The rank order from lowest to highest correlational strength is: dark blue → light blue → green → yellow → orange → red.

Correlational tractography^18^ was derived to visualize pathways that have DTI_FA correlated with duration. A nonparametric Spearman correlation was used to derive the correlation. The statistical significance of the correlation was examined using a permutation test.^19^ A total of 60 subjects were included in the analysis. A T-score threshold of 2.5 was assigned in the fibre tracking algorithm. A seeding region was placed in the whole brain. The tracks were filtered by topology-informed pruning^20^ with 16 iteration(s). An FDR threshold of 0.05 was used to select tracks. To estimate the FDR, a total of 4000 randomized permutations were applied to the group label to obtain the null distribution of the track length (Fig. 5).

## Results

### Demographical and clinical comparisons

As shown in Table 1, the three groups were well-matched in demographic and baseline clinical characteristics, including age (*F* = 1.74, *P* = 0.18), sex (χ^2^ = 0, *P* = 1.00), pain side distribution (*χ*^2^ = 0.58, *P* = 0.45), disease duration and anxiety/depression scores (all *P* > 0.05). While preoperative pain intensity was comparable between groups (*P* = 0.47), postoperative pain outcomes diverged dramatically. The effective group achieved complete pain relief (VAS score from 10 to 0) immediately after surgery, and all patients in this group remained pain-free without recurrence at the 2-year follow-up. In stark contrast, the ineffective group maintained moderate-to-severe pain (VAS 4-9) at the 3-month assessment. Given the persistent pain, all patients in the ineffective group subsequently underwent percutaneous balloon compression (PBC) of the trigeminal nerve within six months post-MVD, as a salvage therapy. This clear dichotomy in long-term outcome and treatment trajectory underscores the fundamental difference between the two groups. Additionally, the haemodynamic parameter (pressure, Pa), reflecting trigeminal microvascular pressure, was significantly higher in the E group (*P* = 0.02). The distribution of the offending vessels responsible for NVC was meticulously recorded during surgery. The superior cerebellar artery (SCA) was the most common culprit across both patient groups. There was no statistically significant difference in the distribution of offending vessel types [SCA, anterior inferior cerebellar artery (AICA), venous compression or combined vessel types] between the effective (E) and non-effective (NE) outcome groups (*P* > 0.05, *χ*² test). This indicates that the type of compressing vessel was not a primary confounding factor underlying the differences in surgical outcomes observed in this cohort.

### Permutation test results for functional connectivity analysis

We first employed a whole-brain, connection-wise permutation test to identify differences in cortico-subcortical functional connectivity between the effective (E) and ineffective (NE) outcome groups across all 1600 connections. While uncorrected analyses revealed a number of connections showing group differences (permutation test: 141 connections with *P* < 0.05; *t*-test: 69 connections with *P* < 0.05), none survived rigorous FDR or FWE correction.

This pattern—widespread uncorrected differences but no significant connections after stringent correction—is common in high-dimensional neuroimaging studies with moderate sample sizes. Rather than reflecting an absence of biological effect, it likely reflects the spatially distributed, moderate-effect-size nature of the connectivity differences between outcome groups. Traditional mass-univariate thresholding may be underpowered to detect such distributed alterations.

Therefore, to identify the most clinically informative features while maintaining statistical rigour, we adopted an effect-size-driven approach. All 1600 connections were ranked by the magnitude of group difference (Cohen’s *d*), and the top-ranked connections were prioritized for detailed characterization and subsequent machine learning analysis. The maximum effect size observed was |*d*| = 1.003, a large effect by conventional benchmarks (|*d*| ≥ 0.8). This effect-size-based strategy allows for the discovery of potentially replicable signatures of poor surgical outcome without exclusive reliance on null hypothesis significance testing in this discovery cohort.

### Top 10 connections by effect size

Based on the statistical results, this study compared the effect sizes (Cohen’s *d*) of cortical-subcortical functional connectivity between the experimental group (E group) and the non-experimental group (NE group), with reference data from a HC group included (Fig. 2 and Table 2). The main findings are as follows.

**Table 2:** Top 10 cortico-subcortical connections by effect size (E group versus NE group)

Rank	Cortical region	Subcortical region	Cohen’s *d*	*P*-value	Mean diff	Direction	Effect size
1	LH_DorsAttn_Post_3	PUT-rh	−1.003	0.000265	−0.12252	E < NE	Large
2	RH_Default_PFCdPFCm_2	AMY-lh	−0.772	0.004086	−0.08201	E < NE	Medium
3	RH_DorsAttn_Post_3	GP-rh	−0.765	0.004421	−0.09014	E < NE	Medium
4	RH_Default_pCunPCC_1	CAU-lh	−0.756	0.004887	−0.08342	E < NE	Medium
5	RH_Vis_1	pTHA-rh	−0.750	0.005168	−0.09602	E < NE	Medium
6	RH_SomMot_3	GP-lh	−0.741	0.005699	−0.08404	E < NE	Medium
7	RH_SomMot_7	CAU-lh	−0.741	0.005725	−0.10808	E < NE	Medium
8	LH_Vis_9	CAU-lh	−0.740	0.005776	−0.08013	E < NE	Medium
9	LH_Vis_4	NAc-rh	−0.740	0.005781	−0.08739	E < NE	Medium
10	LH_Default_PFC_3	AMY-rh	−0.723	0.006907	−0.11855	E < NE	Medium

### Analysis of the connection with maximum effect size

The connection exhibiting the largest effect size was between the cortical region ‘7Networks_LH_DorsAttn_Post_3’ and the subcortical right putamen (PUT-rh), with a Cohen’s *d* of −1.003 (*P* = 0.000265), representing a large negative effect (E < NE). Detailed comparisons showed that the mean connectivity strength for this connection was 0.1391 (SD = 0.1114) in the E group and 0.2617 (SD = 0.1320) in the NE group, resulting in a mean difference (E-NE) of −0.1225. Notably, the mean value for the HC group was 0.2353 (SD = 0.1689), which was lower than the NE group’s mean, suggesting a graded pattern where E < HC < NE in connectivity strength for this specific circuit.

### Top 10 connections by effect size and healthy control group position

All top 10 connections ranked by the absolute value of Cohen’s *d* showed negative effect directions (E < NE), with effect sizes ranging from −0.723 to −1.003, all reaching statistical significance (*P*-values between 0.0003 and 0.0069). These involved cortical regions primarily from the Default, Visual, Dorsal Attention and Somatomotor networks, interacting with subcortical structures including the putamen (PUT), amygdala (AMY), globus pallidus (GP), caudate (CAU), posterior thalamus (pTHA) and nucleus accumbens (NAc).

Importantly, an examination of the HC group’s connectivity coefficients relative to the E and NE groups across these key connections reveals a consistent trend: in the majority of these high-effect-size connections, the mean connectivity strength of the HC group was positioned between that of the E and NE groups, but crucially, it was most often lower than the NE group. For example, in the representative connection involving the right somatomotor network (RH-SomMot, as illustrated in Fig. 2B), the HC group exhibited values that were intermediate but consistently lower than the NE group, while the E group showed the lowest values. This pattern suggests a potential continuum or gradient of connectivity strength across groups: HC values frequently fell below NE but above E, placing the HC group in an intermediate position rather than at the high end of the spectrum.

### Stability of top connections

To assess whether the top 10 connections were robust to individual subject influence, we performed a leave-one-out sensitivity analysis. Across all 60 iterations, the average retention rate was 81.7% for remaining in the top 10% and 98.3% for remaining in the top 15 (Fig. 2C). Notably, the four highest-ranked connections—including key DMN–limbic circuits—remained in the top 10 in over 93% of iterations, demonstrating exceptional stability. While connections ranked 5–10 showed greater variability (range: 40.0–86.7% top-10 retention), all 10 connections remained in the top 15 in at least 83% of iterations (98.3% average). These results confirm that the identified connectivity features reflect stable group differences rather than being driven by outliers.

### Network-level analysis

Network-level statistics indicated that the Default network contained the highest mean absolute effect size (mean |*d*| = 0.218, Fig. 3A) and the highest number of significant connections (27 out of 384, 7.0%, Fig. 3B). The Visual, Dorsal Attention and Somatomotor networks also showed notable proportions of significant connections (3.8% to 5.5%). The Control and Salience/Ventral Attention networks had the lowest proportions of significant connections (1.0% each). Analysis of effect direction revealed that across all networks, connections with stronger connectivity in the NE group (E < NE) outnumbered those with stronger connectivity in the E group (E > NE) (Fig. 3C).

### Effect direction and healthy control reference

Overall, 42.3% of connections showed positive effects (E > NE), while 57.8% showed negative effects (E < NE). Among the 69 statistically significant connections, this pattern was more pronounced: 79.7% (55 connections) were negative (E < NE), and only 20.3% (14 connections) were positive (E > NE). The systematic positioning of the HC group's connectivity values—typically lower than NE but higher than E—provides critical context. This pattern underscores that the NE group does not merely represent a ‘healthier’ state; instead, it exhibits hyperconnectivity relative to the HC baseline in many of the circuits that are hypo-connected in the E group.

### The prediction model

To evaluate the multivariate discriminative potential of the identified cortico-subcortical connectivity differences, a SVM classification analysis was conducted using the top 10 connections ranked by effect size as input features. The classification model was trained and evaluated through 100 repetitions of 5-fold cross-validation to ensure robust performance estimation. The model achieved an average classification accuracy of 86.0% (±0.034 SD) and an average AUC of 0.931 (±0.017 SD), validating the reliability of these functional connectivity features for distinguishing surgical outcomes (Fig. 4).

### DTI results

Correlational tractography showed a significant negative correlation between FA in these tracts and disease duration (FDR <0.05, Fig. 5). This indicates a potential link between the duration of TN and microstructural damage to these tracts, including demyelination and axonal injury. Notably, age and disease duration were not correlated in our sample (Pearson’s *r* = 0.032, *P* = 0.808), confirming that patients with longer disease duration were not systematically older. This indicates that the observed negative correlation between FA and disease duration is unlikely to be confounded by normal ageing processes.

## Discussion

Our study provides three principal findings that collectively redefine the relationship between peripheral pathology, central reorganization and surgical outcome in TN.

First, patients with poor MVD outcome exhibit marked hyperconnectivity between the DMN and limbic structures—a pattern that positions the ineffective-outcome group at the extreme end of a spectrum, with HCs intermediate and effective-outcome patients at the lowest connectivity level. Second, this functional signature predicts surgical outcome with high accuracy (86.0%, AUC = 0.931) when used as input to a multivariate classifier, demonstrating that preoperative connectivity patterns contain clinically actionable information. Third, these functional alterations coexist with progressive microstructural decline in sensorimotor white matter tracts, revealing a paradoxical structure–function dissociation that distinguishes the ineffective-outcome group from both effective-outcome patients and HCs.

Together, these findings challenge a purely peripheral model of TN and invite a fundamental reconceptualization of what constitutes treatment failure—not as ineffective decompression, but as a failure of disease model.^9,10^

1. From sensory signal to self-narrative: DMN–Limbic hyperconnectivity as pain embedding

The pattern of hyperconnectivity observed in the NE group—particularly between the DMN and the amygdala—is not adequately captured by the term ‘central sensitization’. Central sensitization describes enhanced gain in nociceptive pathways^9^; what we observe is enhanced coupling between pain and the neural architecture of the self.^21,22^ The DMN, encompassing medial prefrontal cortex (mPFC), posterior cingulate cortex (PCC) and angular gyrus, is the neurobiological substrate of self-representation, mediating autobiographical memory, mental simulation and self-referential thought.^22,23^ When this system becomes persistently coupled with limbic structures—the amygdala, which encodes threat and emotional salience,^24,25^ the hippocampus, which provides contextual and episodic memory,^26^ and the nucleus accumbens, which mediates motivational salience^27^—pain undergoes a functional transition: it is no longer a sensory event that the patient experiences, but has become functionally embedded within the patient's sense of self.

At a molecular level, this embedding may reflect maladaptive synaptic plasticity within these circuits. Chronic nociceptive input can induce long-term potentiation (LTP)-like changes at amygdala synapses, enhancing output to prefrontal cortical regions.^25^ Concurrently, microstructural decline in sensorimotor white matter tracts—evidenced by our DTI findings—may disrupt local inhibitory circuits, particularly GABAergic interneurons that normally restrain DMN–limbic coupling.^28^ The resulting disinhibition could drive the pathological hyperconnectivity we observe, transforming an adaptive interoceptive signal into a persistent, self-referential pain narrative. This framework positions ‘pain embedding’ as a circuit-level pathology with a plausible molecular substrate, opening avenues for targeted neuromodulation.

We term this phenomenon ‘pain embedding’—the integration of nociceptive signals into the default-mode narrative of the self. Once embedded, pain acquires functional autonomy: it persists even after complete removal of its peripheral trigger, because it is no longer maintained by nociceptive input, but by the same neural circuits that sustain self-awareness and autobiographical memory.

This framework explains the otherwise paradoxical clinical observation that defines our NE group: the surgeon successfully decompresses the nerve, but cannot decompress the patient’s sense of self. The pain persists not because the surgery failed, but because the neural representation of pain has been relocated from the sensory periphery to the cognitive-affective core of the self.

Critically, the positioning of the HC group—consistently intermediate between the E and NE groups across the top-ranked hyperconnected circuits—supports a threshold model of pain embedding. Moderate DMN–limbic coupling may be normative, reflecting adaptive integration of interoceptive signals into self-awareness. However, once a critical threshold is crossed, pain becomes ‘over-embedded’: it dominates the self-narrative, resists extinction and becomes refractory to peripheral intervention. The NE group has crossed this threshold; the E group has not.

This conceptual shift carries direct therapeutic implications. For patients with the ‘pain-embedded’ phenotype, MVD alone is mechanistically misaligned—it addresses the original trigger, but not the current driver, of pain. Effective treatment requires dis-embedding pain from the self-representational system, which may necessitate neuromodulatory interventions—such as repetitive transcranial magnetic stimulation (rTMS) targeting dorsomedial prefrontal cortex or other DMN hubs—to downregulate pathological DMN–amygdala coupling. Such circuit-based interventions could be delivered preoperatively to ‘dis-embed’ pain before decompression or postoperatively as salvage therapy for patients identified by the fMRI biomarker reported here.^29,30^

2. Maladaptive plasticity: structure–function dissociation as a signature of irreversible centralization

A conventional interpretation of the coexistence of high functional connectivity and low white matter integrity would invoke compensatory plasticity: the brain recruits alternative pathways to maintain function in the face of structural decline.^31,32^ Our data argue against this view.

In the NE group, higher functional connectivity was not associated with better clinical status, but with poorer surgical outcome. This is not compensation; it is maladaptive release. We propose that microstructural decline in sensorimotor tracts—evidenced by the negative correlation between FA and disease duration—disrupts local inhibitory circuits, leading to a loss of network restraint rather than adaptive reorganization.

This interpretation is consistent with emerging evidence that chronic pain states are characterized by reduced GABAergic inhibition in both cortical and subcortical structures.^28^ Structural damage to white matter tracts may compromise the integrity of inhibitory projections, resulting in disinhibited connectivity: hyperconnectivity that reflects failure of suppression, not enhancement of function.

We term this phenomenon ‘disinhibited connectivity’ to distinguish it from compensatory plasticity on two key dimensions. First, directionality: compensatory plasticity improves or maintains function; disinhibited connectivity impairs it. Second, reversibility: compensatory plasticity is often reversible upon restoration of input; disinhibited connectivity, once established, may represent a structural–functional scar that persists independently of its original cause.

This framework provides a parsimonious explanation for the otherwise puzzling dissociation between our fMRI and DTI findings. The same patients who have lost white matter integrity in sensorimotor pathways exhibit the highest functional connectivity in DMN–limbic circuits—not because they have successfully compensated, but because inhibitory control over these circuits has been eroded by progressive microstructural damage.

The clinical implication is sobering: patients with longer disease duration and lower FA may have already passed a ‘surgical window’ beyond which peripheral decompression alone cannot reverse central reorganization. Preoperative DTI may serve not only as a marker of microstructural health, but as a chronometric biomarker that estimates how far pain has progressed along the peripheral–central axis.^12,33^

3. The vascular illusion resolved: central connectivity profiles identify a non-compressive phenotype

Our haemodynamic data confirm that a subset of TN patients—those with poor MVD outcomes—exhibit significantly lower vascular pressure despite radiographically unambiguous neurovascular contact. This finding challenges a foundational assumption of TN pathophysiology: that all radiographically visible neurovascular contacts are functionally pathogenic.^5^

We propose the term ‘vascular illusion’ to describe this phenomenon: an anatomical contact that appears compressive on imaging but lacks functional pathogenicity. In these patients, the true driver of pain is not the offending vessel, but the central pain state that has developed independently—or was triggered only transiently—by the contact.

Crucially, this central state is detectable before surgery. The same cortico-limbic hyperconnectivity that predicts poor MVD outcome also identifies patients with low-pressure vascular contact. The fMRI biomarker and the haemodynamic biomarker converge on the same biological phenomenon: pain that has left the periphery and taken residence in the self.

This convergence resolves a long-standing paradox in the TN literature: why do some patients with ‘severe’ compression on imaging have excellent outcomes, while others with ‘mild’ compression fail to improve? The answer, we propose, lies not in the inadequacy of current imaging protocols, but in the misclassification of anatomical contact as pathological compression.

The distinction between ‘contact’ and ‘compression’ is not merely semantic. Contact is an anatomical description; compression is a functional inference. Our data demonstrate that this inference is fallible in a substantial subset of patients—precisely those who fail to benefit from MVD.

This framework transforms the preoperative clinical question from ‘Is there vascular contact?’ to ‘Is this contact pathogenic?’. The answer, we argue, cannot be read from higher-resolution imaging of the nerve. It must be sought in the brain. Preoperative fMRI of cortico-limbic connectivity provides a direct readout of pain centralization status—and, by extension, a functional assay of the pathogenicity of any given vascular contact.

We therefore propose that preoperative resting-state fMRI should be considered not as a research tool, but as a triage instrument to distinguish patients with true compressive pathology (who are likely to benefit from MVD) from those with vascular illusion and established pain embedding (who may require primary or adjunctive central neuromodulation).^26,29,30^

4. Limitations and future directions

Several limitations warrant consideration. First, the cross-sectional design precludes causal inference regarding the temporal sequence of vascular compression, microstructural decline and functional hyperconnectivity. Longitudinal studies beginning at disease onset—though logistically challenging—are needed to determine whether pain embedding represents a progressive complication of chronic nociceptive input or a pre-existing vulnerability that determines surgical outcome independent of disease duration.^12,34^

Second, despite rigorous matching on demographic and clinical variables, residual confounding by unmeasured factors—including genetic polymorphisms affecting sodium channel function,^35^ subclinical trigeminal nerve morphological variations and psychosocial contributors such as catastrophizing or trauma history—cannot be excluded. Multimodal studies integrating genomics, high-resolution structural imaging and comprehensive neuropsychological assessment are needed to fully characterize the ‘pain-embedded’ phenotype.

Third, the SVM classifier, while robust in internal cross-validation, requires external validation in independent, multi-centre cohorts with diverse scanner platforms and surgical protocols. The generalizability of the top 10 connectivity features identified in this discovery cohort remains to be established.

Fourth, while our leave-one-out sensitivity analysis confirmed the stability of the top-ranked connectivity features, the variability observed in lower-ranked connections (ranks 5–10) suggests that a degree of uncertainty remains in feature selection. External validation in independent cohorts will be necessary to establish the generalizability of the specific connectivity pattern identified here.

Fifth, while our DTI analysis revealed a significant negative correlation between FA and disease duration—and age was not confounded with disease duration in our sample (*r* = 0.032, *P* = 0.808)—the absence of diffusion data from HCs limits interpretation of this finding. Without an age-matched control group, we cannot definitively determine whether the observed FA decline exceeds age-expected trajectories. Future longitudinal studies incorporating both patients and age-matched HCs with diffusion imaging are needed to establish whether the microstructural changes observed here represent true disease-related pathology beyond normal ageing.

Sixth, while our haemodynamic data revealed significantly lower vascular pressure in the NE group—supporting the concept of ‘vascular illusion’—the relationship between individual haemodynamic profiles and DMN–limbic connectivity could not be directly examined due to the limited subsample with complete CFD data. Whether low-pressure vascular contact represents a true incidental finding or interacts with central pain states through more complex mechanisms remains an open question. Future studies with larger samples and integrated haemodynamic–functional imaging protocols are needed to directly test the coupling between peripheral vascular dynamics and central pain circuitry.

Finally, the concept of ‘pain embedding’—though empirically grounded in the specific connectivity pattern we report —requires prospective validation as a predictive biomarker. Such validation could integrate multimodal data including functional connectivity (DMN–limbic coupling), structural imaging (white matter integrity), behavioural measures (pain catastrophizing, self-referential thought) and task-based fMRI to fully characterize the ‘pain-embedded’ phenotype. A clinical trial that randomizes patients with high versus low DMN–limbic connectivity to MVD alone versus MVD plus neuromodulation would provide the strongest test of the framework proposed here.^29,30^

## Conclusions

This study demonstrates that preoperative cortico-limbic hyperconnectivity is not merely a correlate of poor surgical outcome—it is the neural signature of a distinct disease phenotype. We propose that a substantial subset of TN patients suffer not from peripheral compression, but from a central network disorder in which pain has become functionally embedded within the brain’s default-mode representation of the self.

For these patients, MVD fails not because the surgery was technically inadequate, but because the surgeon removes the compression, yet cannot remove the pain from the patient’s sense of self. This is not a failure of surgical skill, but a failure of disease model.

We therefore advocate for a reclassification of TN along a peripheral–central axis, rather than a purely anatomical one. Patients with the ‘pain-embedded’ phenotype should be stratified not by the presence or severity of vascular contact, but by their central pain self-representation status. Future treatment paradigms should integrate preoperative fMRI-guided phenotyping and circuit-based neuromodulation—for instance, rTMS targeting DMN nodes such as the medial prefrontal cortex to attenuate amygdala hyperconnectivity—to ‘unlearn’ the central pain trace—either alongside or instead of surgical decompression.^26,29,30^

The future of TN surgery lies not in better visualization of the nerve, but in better understanding of the brain that perceives it.

## Data Availability

The datasets used and/or analysed during the current study are available from the corresponding author on reasonable request. All custom MATLAB scripts used for data analysis have been deposited in Zenodo (DOI:10.5281/zenodo.19106465) and are publicly available at https://zenodo.org/records/19106465.
